# Resting-state functional connectivity of ventral parietal regions associated with attention reorienting and episodic recollection

**DOI:** 10.3389/fnhum.2013.00038

**Published:** 2013-02-22

**Authors:** S. M. Daselaar, W. Huijbers, K. Eklund, M. Moscovitch, R. Cabeza

**Affiliations:** ^1^Department of Psychology and Neuroscience, Duke UniversityDurham, NC, USA; ^2^Donders Institute for Brain, Cognition and Behaviour, Radboud University NijmegenNijmegen, Netherlands; ^3^Martinos Center for Biomedical Imaging, Harvard Medical School, Brigham and Women's HospitalBoston, MA, USA; ^4^Department of Psychology, University of TorontoON, Canada

**Keywords:** bottom-up attention, episodic memory, functional connectivity, resting state fMRI, ventral parietal cortex

## Abstract

In functional neuroimaging studies, ventral parietal cortex (VPC) is recruited by very different cognitive tasks. Explaining the contributions of VPC to these tasks has become a topic of intense study and lively debate. Perception studies frequently find VPC activations during tasks involving attention-reorienting, and memory studies frequently find them during tasks involving episodic recollection. According to the Attention to Memory (AtoM) model, both phenomena can be explained by the same VPC function: bottom-up attention. Yet, a recent functional MRI (fMRI) meta-analysis suggested that attention-reorienting activations are more frequent in anterior VPC, whereas recollection activations are more frequent in posterior VPC. Also, there is evidence that anterior and posterior VPC regions have different functional connectivity patterns. To investigate these issues, we conducted a resting-state functional connectivity analysis using as seeds the center-of-mass of attention-reorienting and recollection activations in the meta-analysis, which were located in the supramarginal gyrus (SMG, around the temporo-parietal junction—TPJ) and in the angular gyrus (AG), respectively. The SMG seed showed stronger connectivity with ventrolateral prefrontal cortex (VLPFC) and occipito-temporal cortex, whereas the AG seed showed stronger connectivity with the hippocampus and default network regions. To investigate whether these connectivity differences were graded or sharp, VLPFC and hippocampal connectivity was measured in VPC regions traversing through the SMG and AG seeds. The results showed a graded pattern: VLPFC connectivity gradually decreases from SMG to AG, whereas hippocampal connectivity gradually increases from SMG to AG. Importantly, both gradients showed an abrupt break when extended beyond VPC borders. This finding suggests that functional differences between SMG and AG are more subtle than previously thought. These connectivity differences can be explained by differences in the input and output to anterior and posterior VPC regions, without the need of postulating markedly different functions. These results are as consistent with integrative accounts of VPC function, such as the AtoM model, as they are with models that ascribe completely different functions to VPC regions.

## Introduction

Located ventrally to the intraparietal sulcus, the ventral parietal cortex (VPC) is comprised of the supramarginal gyrus [SMG (BA40)] and angular gyrus [AG (BA39)]. In line with traditional views linking the parietal cortex to attention (Mesulam, 1981), there is abundant functional MRI (fMRI) evidence of the involvement of VPC in attentional reorienting to sensory stimuli outside the immediate focus of attention (Corbetta and Shulman, 2002; Corbetta et al., 2008). At the same time, there are abundant fMRI data linking VPC to vivid episodic recollection (Ciaramelli et al., 2008; Vilberg and Rugg, 2008). For example, VPC shows greater activity for items remembered with than without contextual details and for items with high than low confidence (Henson et al., 1999; Eldridge et al., 2000; Wheeler and Buckner, 2004; Yonelinas et al., 2005; Daselaar et al., 2006). According to the Attention to Memory (AtoM) model, the involvement of VPC in attention-reorienting during perception tasks and in recollection during memory tasks reflect the same underlying cognitive function: bottom-up attention (Cabeza et al., 2008). This model defines bottom-up attention as attention captured by information entering working memory. Since the latter includes not only incoming sensory information but also information retrieved from long-term memory, this model assumes that bottom-up attention can be captured not only by salient external stimuli but also by vivid memories. Thus, the AtoM model provides a parsimonious account for VPC activations in perception and memory domains, as well as in other cognitive domains (Cabeza et al., 2012).

**Figure 1:**
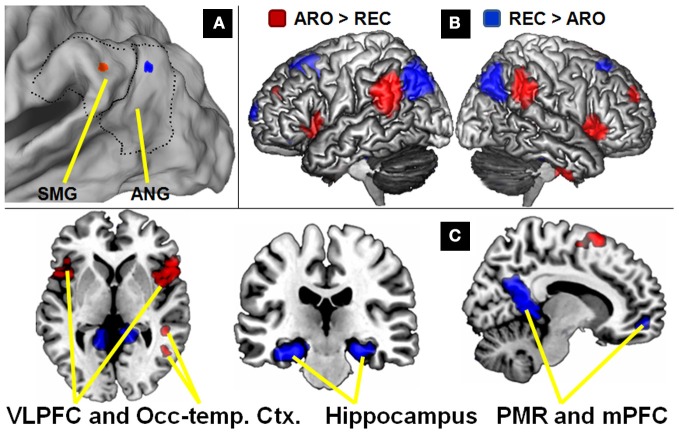
**Differences in connectivity between *attention-reorienting* and *recollection* seeds. (A)** VPC coordinates derived from meta-analyses of *attention-reorienting* and *recollection* plotted on a 3D rendering. Dotted lines indicate the boundaries of VPC, and the solid black line in the middle the distinction between supramarginal gyrus (SMG) and angular gyrus (AG). **(B)** Comparison of the two seeds plotted on 3D renderings of the left and right sides of the brain, illustrating the hemispheric symmetry of the findings. Although left-sided seeds were used, results are virtually identical in the right hemisphere. **(C)** Regions showing greater VPC connectivity for the *attention-reorienting* (red) and *recollection* (blue) seeds plotted on 2D slices. Examples of regions showing greater VPC connectivity for the *attention-reorienting* seed include left and right ventrolateral PFC, and occipito-temporal regions. For the *recollection* seed, they include bilateral hippocampus, and the “default mode network” regions (PCC, Posterior cingulate cortex; mPFC, medial prefrontal cortex).

However, the AtoM model's integrative account of VPC function has been recently challenged by a meta-analysis of fMRI studies that reported a difference in the spatial distribution of VPC activations during attention-reorienting and recollection tasks (Hutchinson et al., 2009). According to this meta-analysis, the center-of-mass of VPC activations during attention-reorienting tasks is located in anterior VPC, within the SMG, whereas the center-of-mass of VPC activations during recollection tasks is located in posterior VPC, within the AG (Corbetta et al., 2008; Hutchinson et al., 2009). These two locations are displayed in Figure 1A. This finding is not necessarily incompatible with the AtoM model because this model assumes that VPC contributes similar bottom-up attention processes to attention-reorienting and recollection tasks, not that the locations of VPC activations in these two tasks must be identical. Also, fMRI meta-analyses based on activation peaks do not consider the spatial extent of activations in individual studies and hence cannot determine whether activations for different conditions do or do not overlap. Moreover, the results of fMRI meta-analyses are confounded by many possible differences in methods and participants among the studies surveyed.

To investigate these issues, a recent fMRI study compared the distribution of VPC activations during attention-reorienting and recollection conditions directly within-participants (Cabeza et al., 2011). In the attention-reorienting condition, participants searched a stream of consonants on the screen and pressed a key when they detected a vowel (an oddball task), whereas in a recollection task, they searched their memory for previously studied word-chains and pressed a key when they detected the last word of each chain. Consistent with the AtoM model, conjunction analyses revealed that in both tasks detection (bottom-up attention) activated similar VPC regions in the two tasks. Attention-reorienting and recollection activations overlapped within SMG, but recollection activations were larger and extended more posteriorly toward AG (Hutchinson et al., 2009). These results suggest that attention-reorienting and recollection activations can overlap within-participants but the distributions may not be identical, which could explain the center-of-mass difference detected in the aforementioned meta-analysis (Hutchinson et al., 2009).

If VPC contributes similarly to bottom-up attention processes in attention-reorienting and recollection tasks, why do the VPC activations they elicit show some differences in spatial distribution? Why do recollection activations tend to extend more posteriorly toward AG than attention-reorienting activations? One possible explanation is that VPC includes intermixed neuronal populations that differ in connectivity with other brain regions. If this is the case, the exact locations of fMRI activations are likely to vary depending on the demands that specific cognitive tasks place on VPC interactions with other brain regions, both in terms of inputs and outputs. Regarding inputs to VPC, if anterior VPC regions have stronger connectivity with visual cortex that would explain more anterior activations during attention-reorienting tasks, which typically involve visuospatial stimuli (Hutchinson et al., 2009). Conversely, if posterior VPC regions have stronger connectivity with medial temporal lobe (MTL) regions, that would explain more posterior activations during recollection tasks, which depend on input from MTL memory regions, such as the hippocampus. Regarding outputs from VPC, if anterior VPC regions have stronger connectivity with premotor frontal regions (Kelly et al., 2010), that would explain more anterior activations during attention-reorienting tasks, which typically require speeded responses. On the other hand, if posterior VPC regions have stronger connectivity with the default mode network (DMN) regions associated with the construction of internal scenarios (Buckner et al., 2008), that could explain more posterior activations during recollection tasks, which usually require the creation of such internal scenarios before a response can be made. Preliminary evidence that these hypothetical differences in connectivity do exist has been provided by recent resting-state functional connectivity studies (Vincent et al., 2008; Nelson et al., 2010; Uddin et al., 2010; Yeo et al., 2011). For example, these studies found that more anterior VPC regions have stronger connectivity with ventrolateral PFC (VLPFC), whereas more posterior VPC regions have stronger connectivity with MTL and DMN regions.

However, there are two problems in using this functional connectivity evidence as evidence in favor of our input-output hypothesis. First, given that the anatomical borders within VPC are still relatively ill-defined (Uddin et al., 2010), it is unclear to what extent the anterior and posterior VPC regions investigated in previous resting state connectivity studies (Vincent et al., 2008; Nelson et al., 2010; Uddin et al., 2010; Yeo et al., 2011) match with the locations associated with attention-reorienting and recollection tasks in the fMRI meta-analysis (Hutchinson et al., 2009). For example, in one study the anterior VPC regions were in anterior AG rather than in SMG (Uddin et al., 2010). Thus, in order to link attention-reorienting and recollection differences to connectivity differences, it is critical to investigate connectivity differences study using as seeds the actual center-of-mass of attention-reorienting and recollection activations. Second, our output-input hypothesis would be more consistent with anterior-posterior differences in VPC connectivity if these differences are graded than if they are sharp. Sharp differences would fit better with the idea of very different cognitive functions in SMG vs. AG (Nelson et al., 2010). To investigate if anterior-posterior differences in VPC connectivity are graded one needs to sample connectivity in a continuous manner between anterior and posterior VPC regions.

The goals of present study were to address these two problems. First, to link connectivity differences to the distribution of attention-reorienting and recollection activations, we conducted a resting-state functional connectivity using as seeds the center-of-mass of attention-reorienting and recollection activations in the aforementioned fMRI meta-analysis (Hutchinson et al., 2009). Since there is a tendency for recollection studies to show left-sided activations, we used VPC seeds in the left hemisphere only, but explored both left- and right-hemisphere connectivity. Despite the close proximity of the attention-reorienting and recollection seeds (see Figure 1A), we expected differences in functional connectivity patterns. In particular, we predicted that the SMG seed would show stronger connectivity with VLPFC, which is a region previously associated with attention-reorienting (Corbetta and Shulman, 2002), whereas the AG seed would show stronger connectivity with MTL, and in particular with the hippocampus, which is a region previously associated with recollection (Eichenbaum et al., 2007).

Second, we investigated whether functional connectivity differences between the anterior SMG seed and the posterior AG seed are sharp or graded. For this analysis, we focused on VPC connectivity with the two regions most strongly associated with attention-reorienting and recollection, namely VLPFC and the hippocampus, respectively. Instead of a categorical contrast between two VPC locations, as in previous studies, we sampled connectivity continuously between the anterior SMG seed and the posterior AG seed. If one assumes that peak activations in different regions of VPC reflect differences in cognitive function, and that the latter goes hand-in-hand with differences in connectivity, then marked differences in function can be assumed to entail sharp differences in connectivity. Alternatively, these different regions may have a common function, with differences in peak activation reflecting the different inputs and outputs on which that function is applied. If that is the case, then differences in connectivity between the peaks are likely to be graded. In line with our hypothesis, we predicted that differences in VLPFC and hippocampal connectivity would be graded rather than sharp, with VLPFC connectivity decreasing gradually from anterior to posterior regions and hippocampal connectivity showing the opposite trend.

## Methods

### Participants

Resting-state scans were acquired from 24 participants (14 female, mean age 23) recruited from the University of Amsterdam community. All participants were in good health and right-handed. Their native language was Dutch and they were paid 25 euro for participation. Participants gave their written informed consent and the study met all criteria for approval of the ethical board of the Amsterdam Medical Center.

### Scanning

fMRI images were collected on a Phillips Intera 3.0T using a 6-channel standard SENSE head coil and a T2^*^ sensitive gradient echo sequence (96 × 96 matrix, TR 2000 ms, TE 30 ms, FA 80°, 34 slices, 2.3 × 2.3 mm voxel size, 3-mm thick transverse slices). Additionally, a high-resolution T1-weighted structural scan (256 × 256 matrix, TR 12 ms, TE 5 ms, FOV 24 cm, 68 slices, 1 mm slice thickness) was collected. Two 8-min rest blocks were administered to each participant. Each block consisted of a black screen with a white fixation cross-hair in the center. Participants were instructed to keep focused on the cross-hair during scanning.

### Image preprocessing

Statistical Parametric Mapping (SPM5; http://www.fil.ion.ucl.ac.uk/spm) software was used to preprocess and analyze the MR data. The images were slice-time and motion-corrected, and then normalized. First, individual normalization parameters were obtained by normalizing the segmentedstructural scan of each subject using the Montreal Neurological Institute (MNI) T1 template image. These normalization parameters were then applied to the functional images. Next, the normalized functional images were resliced to a resolution of 3 × 3 × 3 mm and spatially smoothed using an 8-mm isotropic Gaussian kernel. Next, treating the volumes as a time series, the data were temporally smoothed using linear detrending, and a temporal filter (0.01 Hz < *f* < 0.08 Hz) was applied to remove low-frequency drifts and physiological high-frequency noise.

### VPC seeds

For the identification of the seeds, we used the center-of-mass of *attention-reorienting* activations in an fMRI meta-analysis (Hutchinson et al., 2009), which also reported the center-of-mass of *recollection* activations previously estimated by other reviews (Ciaramelli et al., 2008; Vilberg and Rugg, 2008). In the present study, we used the MNI coordinate system. Since the metaanalysis also included studies reporting their results in Talairach space, we first converted these coordinates to MNI space (Brett et al., 2001). Then we calculated the center-of-mass using the median of the resulting *x*, *y*, and *z* coordinates for both *attention-reorienting* and *recollection* studies. For the *attention-reorienting* studies (*N* = 23) the center of mass was *x*, *y*, *z* = −48, −45, 34, for *recollection* studies (*N* = 20), it was *x*, *y*, *z* = −43, −58, 36, (Vilberg and Rugg, 2008). Figure 1A shows a rendering of VPC including the boundaries between SMG and AG using the CARET software package (http://brainvis.wustl.edu/wiki/index.php/Caret:About). As can be seen, the *attention-reorienting* seed (red dot) was located more anteriorly, falling within SMG (BA 40) in a region commonly referred to as the temporo-parietal junction (TPJ) (Corbetta et al., 2008). In contrast, the *recollection* seed (blue dot) was located more posteriorly falling within the boundaries of AG (BA 39).

### Functional connectivity analyses

#### Connectivity differences between SMG vs. AG seeds

For analysis of the connectivity differences between the SMG and AG seeds we used the REST toolbox (V1.3; www.restfmri.net) integrated with SPM5. A seed correlation analysis was performed in a voxel-wise way with the six head motion parameters as covariates. Individual *r*-maps were normalized to *Z*-maps by using Fisher's *Z* transformation. For the seed analysis, two spherical regions of interest (ROIs; radius = 6 mm) were centered at the MNI coordinates derived from the two meta-analyses of *attention-reorienting* and *recollection*. Next, the *Z*-maps were averaged across runs for each participant. Subsequently, difference maps were created by subtracting the two resulting *Z*-maps. To assess relative differences in connectivity between the two seeds, we used a random effects analysis on these difference maps with a two-sided one-sample *t*-test (*P* < 0.001, minimum cluster size = 25).

#### Gradedness

**Figure 2:**
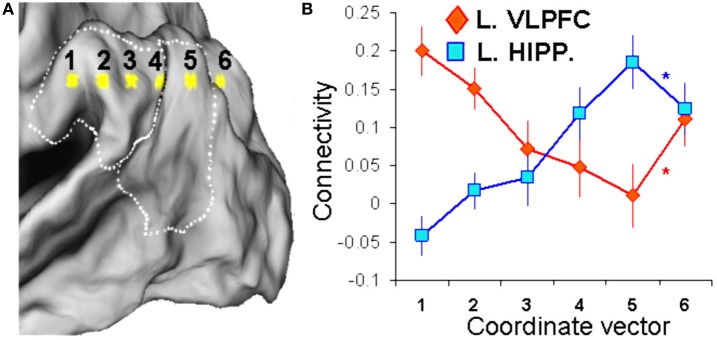
**Graded differences between *attention-reorienting* and *recollection* seeds. (A)** Depiction of a 3D vector consisting of 6 points (6 mm apart) traversing through the *attention-reorienting* and *recollection* VPC seeds, which was used to investigate the VPC connectivity patterns with VLPFC and hippocampus. **(B)** Line graphs are depicting VPC connectivity for VLPFC (red line) and hippocampus (blue line) along the 3D vector. Results show that both VLPFC and hippocampal connectivity showed a graded pattern along the first five points of the vector that fell within VPC, but a sharp change when the vector exits the VPC boundaries and continues into parieto-occipital cortex (BA 19; point 6). These findings indicate that connectivity differences between the *attention reorienting* and *recollection* VPC seeds are more graded than discrete.

To assess whether differences in connectivity between the SMG and AG seeds were sharp or graded, we used a four-step approach. First, to avoid spatial overlap, we reduced the smoothing kernel of the resting-state scans from 8 to 4 mm. Second, we created a directional 3D vector by subtracting the *attention-reorienting* and *recollection x, y, z* seed coordinates. This vector was then extended to cover 6-points (point 1 *x*, *y*, *z* = −54, −34, 35; point 6 *x*, *y*, *z* = −30, −79, 35) with 2 intermediate voxels (6-mm). As shown in Figure 2A, this vector included VPC as well as one point beyond the borders of VPC that fell into parieto-occipital cortex (BA19). We did not apply the same 4 mm smoothing kernel in the main analysis (standard 8 mm kernel), because this analysis was used to find connectivity differences between the two adjacent VPC seeds. Identifying reliable regions showing differences in connectivity across the whole-brain requires more statistical power than the regionally-focused gradient analyses. Third, we defined the two regions most clearly linked in the literature to *attention-reorienting* (Corbetta et al., 2008) and *recollection* (Eichenbaum et al., 2007), VLPFC and hippocampus, as ROIs within the left hemisphere. Subsequently, we used the maximum difference in VPC connectivity between *attention-reorienting* and *recollection* seeds within these ROIs (left VLPFC *x*, *y*, *z* = −45, 36, −6; left hippocampus *x*, *y*, *z* = −33, −21, −21; see also Table 1), and then used these locations as seeds in a functional connectivity analysis (same procedures as described above). This step generated two new brain connectivity maps, one for VLPFC, and one for hippocampus. As a fourth step, we examined gradedness by extracting the VLPFC and hippocampal connectivity values from each of the six points of the VPC vector to assess relative differences in VPC connectivity along the vector coordinates.

**Table 1:** **Differences in connectivity between *attention-reorienting* and *recollection* seeds**.

**Region**	**Hemisphere**	***BA***	***X***	***Y***	***Z***	**T attention-reorienting**	**T recollection**
**ATTENTION-REORIENTING > RECOLLECTION**							
Ventrolateral PFC	Left	44/47	−45	36	−6	12.7	7.8
	Right	44/47	51	33	−9	11.1	6.2
Superior frontal Ctx.	Left	8	−24	45	21	5.5	2.5
	Right	8	30	54	30	5.0	1.1
Middle frontal Ctx.	Right	10	12	15	63	5.7	3.0
wOccipito-temporal Ctx.	Right	19	30	−72	12	3.6	2.2
	Right	20	48	−42	6	10.3	6.7
**RECOLLECTION > ATTENTION-REORIENTING**							
Hippocampus (HF)	Left	–	−33	−21	−21	3.0	5.7
	Right	–	30	−27	−21	5.5	8.5
Post. midline region	Left	23/29/30/31	−3	−33	33	12.0	15.1
Medial PFC	Right	10	3	57	−6	6.1	9.5
Frontal pole	Left	10	−15	72	12	5.2	8.4
Superior frontal Ctx.	Left	8	−27	24	51	9.0	11.7
	Right	8	30	27	54	7.8	11.8

*New Hutchinson in semmematt_updated2*.

## Results

### Differences in functional connectivity

Differences in functional connectivity between the SMG and AG seeds are listed in Table 1, and displayed in Figures 1B and C. As shown in Figure 1B, although we used a left-sided seed, the majority of connected regions in the left hemisphere also appear in the right hemisphere. In line with previous research, this finding indicates that there is considerable communication between left and right hemispheres, and that the brain connectivity in the two hemispheres is very similar (Gazzaniga, 2000; Toro et al., 2008). The generality of the findings across hemispheres also indicates that our findings are robust and can not be easily related to physiological noise factors (Birn et al., 2006, 2008). Also, the fact that we directly compared whole-brain correlations for the two seeds should reduce the impact of common noise factors across regions.

As predicted, compared to the AG seed, the SMG seed showed stronger connectivity with bilateral VLPFC regions (see Figure 1C). This finding is consistent with those reported in previous task-based fMRI studies (Corbetta and Shulman, 2002; Corbetta et al., 2008) and with our input/output hypothesis. Interestingly, the AG seed also showed stronger connectivity with temporo-occipital regions (BA19/20) associated with visual processing (Figure 1C). As mentioned before, stronger connectivity between SMG and visual cortex could explain why *attention-reorienting* tasks, which typically involve visuospatial stimuli, tend to engage more anterior VPC regions.

Also consistent with our predictions, compared to the SMG seed, the AG seed showed stronger connectivity with the hippocampus. This region is strongly associated with memory recovery processes which provide the input during *recollection* tasks. The AG seed also showed stronger connectivity with DMN regions such as medial PFC and posterior cingulate cortex (Figure 1C). As noted before, the DMN is involved in constructing internal scenarios (Buckner et al., 2008), which are necessary for the output of *recollection* tasks. Regarding the uncorrected threshold (*p* < 0.001) peaks reported in Table 1, it is important to note that all regions also survived an FDR-correction for multiple comparison at *P* < 0.05 (Nichols and Hayasaka, 2003).

### Graded differences in functional connectivity

To investigate if the connectivity differences between anterior and posterior VPC are graded or sharp, we measured the strength of VLPFC and hippocampal connectivity along a six-point 3D vector stretching throughout VPC, and going through the SMG and AG seeds (Figure 2A). As illustrated by Figure 2B, both VLPFC and hippocampal connectivity showed a graded pattern along the first five points of the vector that fell within VPC, but a sharp change when the vector exits VPC and continues into parieto-occipital cortex (BA 19; point 6). To confirm the observation of a graded pattern within VPC and a sharp change outside the boundaries of VPC, we conducted a two-sided *T*-test comparing the slope differences between consecutive points along the vector gradients across participants, separately for VLPFC and hippocampal connectivity. As indicated by the asterisks in Figure 2B, there were no significant differences in the slopes between the first five neighboring points of the vector gradients, neither for VLPFC connectivity (point 1–2 vs. 2–3: *p* = 0.59; point 2–3 vs. 3–4: *p* = 0.31; point 3–4 vs. 4–5: *p* = 0.82), nor for hippocampal connectivity (point 1–2 vs. 2–3: *p* = 0.87; point 2–3 vs. 3–4: *p* = 0.24; point 3–4 vs. 4–5: *p* = 0.57). However, there was a significant change in slope when exiting VPC (VLPFC, point 4–5 vs. 5–6: *p* = 0.025; hippocampus, point 4–5 vs. 5–6: *p* = 0.016). Thus, our results indeed indicate that differences in connectivity patterns between VPC subregions associated with *attention-reorienting* and *recollection* are more graded rather than sharp, and that this pattern of gradedness is interrupted when extending the vector outside VPC. This last finding indicates that our results cannot easily be explained by confounding aspects associated with preprocessing of the functional images.

## Discussion

In this fMRI study, we compared differences in functional connectivity between two different seeds in left VPC, an anterior seed in SMG corresponding to the center-of-mass of activations during *attention-reorienting* tasks and a posterior seed in AG corresponding to the center-of-mass of activations during *recollection* tasks (Hutchinson et al., 2009). The study yielded two main findings. First, despite their vicinity, the two seeds yielded clear differences in functional connectivity with several brain regions. The SMG seed had stronger connectivity with VLPFC and occipito-temporal regions, whereas the AG seed had stronger connectivity with the hippocampus and DMN regions (Figure 1C). Although we used left-sided seeds, these differences were translated to the right side of the brain, demonstrating the communication between the two hemispheres and the robustness of the findings. Second, a connectivity analysis focusing on VLPFC and hippocampus indicated that the connectivity differences between the SMG and AG seeds are graded, rather than sharp. As one moves from anterior to posterior VPC regions, VLPFC connectivity decreases gradually whereas hippocampal activity increases gradually (Figure 2B). The two findings are discussed in separate sections below.

### Connectivity differences between SMG and AG seeds

A meta-analysis of fMRI studies reported a difference between *attention-reorienting* studies showing more anterior VPC activations and *recollection* studies showing more posterior VPC activations (Hutchinson et al., 2009). However, a study that compared *attention-reorienting* and *recollection* directly within-participants found overlapping activations with some differences in distribution (Cabeza et al., 2011). To harmonize these findings, we proposed an input/output hypothesis: rather than a sharp dissociation in cognitive functions, anterior-posterior differences in the distribution of VPC activations may reflect differences in the typical input and output of *attention-reorienting* and *recollection* tasks. Consistent with this hypothesis, recent resting-state functional connectivity studies have yielded differences in connectivity between anterior and posterior VPC that generally match input/output differences between *attention-reorienting* and *recollection* tasks (Nelson et al., 2010; Uddin et al., 2010; Yeo et al., 2011). Yet, given that anatomical VPC distinctions are still not well determined (Uddin et al., 2010), it was unclear if the VPC locations associated with *attention-reorienting* and *recollection* studies in the fMRI meta-analysis do in fact show significant differences in connectivity. To address this issue, we conducted a resting-state functional connectivity analysis that used as seed the center-of-mass of *attention-reorienting* and *recollection* activations in this meta-analysis. The results confirmed the existence of significant connectivity differences between these seeds: the SMG seed associated with *attention-reorienting* had stronger connectivity with VLPFC and occipito-temporal regions, whereas the AG associated with *recollection* had stronger connectivity with the hippocampus and DMN regions (Figure 1C).

The finding that the SMG seed, which was located in TPJ, showed stronger connectivity with VLPFC is consistent with evidence that TPJ and VLPFC tend to be co-activated in *attention-reorienting* studies (Corbetta and Shulman, 2002; Corbetta et al., 2008). According to Corbetta and Shulman's attention model, TPJ and VLPFC comprise a ventral fronto-parietal network that mediates *attention-reorienting* to stimuli outside the immediate focus of attention (Corbetta and Shulman, 2002; Corbetta et al., 2008). Our data confirms the coupling between TPJ and VLPFC. However, this is the first study showing that the specific TPJ region associated with *attention-reorienting* activations is differentially more connected with VLPFC than the AG region associated with *recollection* activations.

Although we did not have specific predictions regarding laterality, the two VPC seeds clearly revealed a bilateral pattern of connectivity (Figure 2). Regarding the attention-reorienting seed, Corbetta and Shulman's model emphasizes the role of right TPJ in *attention-reorienting*, whereas in the current study the TPJ seed was in the left hemisphere. Nonetheless, this left-lateralized region showed strong connections with both left and right VLPFC regions. The current results suggest that the *attention-reorienting*-related TPJ-VLPFC networks could be more bilateral than previously thought. We took the VPC coordinates from the Hutchinson et al. (2009) meta-analysis, which included many attention-reorienting studies showing left VPC activations, and we used the same approach to identify our seeds. As they mention in the introduction, they focused their meta-analysis on left VPC given that recollection activations tend to be left-lateralized, which might relate to differences in stimulus materials used in attention-reorienting and recollection tasks (Milner, 1971). Most attention studies use non-verbal/meaningless stimuli, such as flashes and sounds, whereas retrieval studies use verbal/meaningful stimuli, such as words. In line with material-specific laterality, a recent episodic retrieval fMRI study that used abstract musical stimuli found old-new effects only in the right parietal cortex (Klostermann et al., 2009). Other studies focusing on the neural correlates of recollection have also found activations only in right VPC (Cansino et al., 2002; Kahn et al., 2004). At the same time, several attention studies show activations only in the left (Mayer et al., 2006; Vossel et al., 2009). In general, most attention-reorienting and recollection studies included in the Hutchinson review have found bilateral VPC activations. There is one study (Guerin and Miller, 2009) that found a retrieval success effect in parietal cortex that showed a tendency for left-lateralized activity compared to attention, regardless of stimulus type (words/faces). However, this study investigated old/new effects, and not specifically recollection. They also did not report an effect in VPC, but in dorsal parietal cortex. Thus, in general and in line with our connectivity data, findings indicate that the lateralization difference in VPC activity between recollection and attention-reorienting studies is more relative than absolute.

In terms of our input/output hypothesis, the strong connectivity of the SMG seed with VLPFC could explain why VPC activations tend to be more anterior for *attention-reorienting* than *recollection*. Most *attention-reorienting* tasks require speeded responses, and VLPFC is directly linked to premotor regions (Kelly et al., 2010) important for such output. In contrast, *recollection* tasks are less dependent on rapid responses. The input/output hypothesis fits also well with the finding that the SMG seed was more strongly connected with visual occipito-temporal regions (BA 19/20) than the AG seed. Most *attention-reorienting* tasks involve visuospatial stimuli and hence emphasize occipito-temporal input to VPC.

Turning to the AG seed associated with *recollection*, the stronger connectivity of this seed with the hippocampal formation is consistent with the role of the well-known role of hippocampus in *recollection*. The AG seed also showed stronger connections with DMN regions, including the posterior cingulate cortex and medial prefrontal cortex. The DMN tends to be more activated during rest than during active task conditions. According to one account, the processes mediated by the DMN represent an *internal mode of cognition* (Buckner et al., 2008; Huijbers et al., 2011), mediating internally oriented processes involving internal reflections, which follow a memory cue or are generated spontaneously. Interpreted in terms of our input/output hypothesis, these functional connectivity results could explain the association between AG and *recollection* tasks. During *recollection* tasks, the input of VPC are long-term memories recovered by MTL and the output involves the construction of an internal scenario mediated by DMN. Thus, because of its input and output characteristics, *recollection* tasks are more likely to engage posterior VPC regions that have stronger connectivity with MTL and DMN regions.

In terms of the relation between our functional connectivity findings and underlying neural processes, it should be noted that we cannot measure direct neural communication with fMRI, but only slow oscillations in brain activity. However, there is substantial evidence indicating that these slow oscillations represent fluctuations in the power of synchronized neuronal activity (Anderson, 2008; Nir et al., 2008; de Pasquale et al., 2010), and that they show correlations with structural brain connectivity measures (Honey et al., 2007; van den Heuvel et al., 2009; Uddin et al., 2010). Moreover, fMRI connectivity measures have been reliably linked to fluctuations in multi-unit neural activity. For instance, a recent brain connectivity study that combined intracortical neurophysiological recording techniques with fMRI (Shmuel and Leopold, 2008) found clear correlations in brain connectivity across visual cortex regions between fMRI measures and multi-unit recordings (spiking rate and power changes). Thus, even though fMRI cannot provide a measure of direct neural communication, it certainly yields insights into the neural coupling between different brain areas, and on a larger spatial scale than can be achieved with electrophysiological techniques.

### Graded connectivity patterns

Although the differences in functional connectivity we found between the SMG and AG seeds were generally consistent with our input/output hypothesis, it is important to determine if these differences are graded or sharp. As noted before, graded differences would fit well with the input/output hypothesis, whereas sharp differences would be more consistent with different cognitive operations in SMG and AG (Hutchinson et al., 2009). To investigate whether connectivity differences were grade or sharp, we sampled connectivity in a continuous manner between the SMG and AG seeds. As illustrated by Figure 2B, the results using critical VLPFC and hippocampal seeds clearly supported a graded pattern: as one moves from anterior to posterior VPC, VLPFC connectivity decreases gradually whereas hippocampal connectivity increases gradually.

The graded connectivity patterns we observed (Figure 2B) are more likely to be associated with a common function mediated by adjacent regions of VPC, as we predicted, than with markedly different functions subserved by these regions which would entail sharp differences in connectivity. Thus, the graded connectivity patterns fit well with the idea that SMG and AG serve a common function mediated by intermixed neuronal populations, whose proportions differ from anterior to posterior VPC regions. Anterior VPC regions have stronger connections with VLPFC, which could explain a more anterior distribution of VPC activations during *attention-reorienting* tasks, whereas posterior VPC regions have stronger connections with the hippocampus, which could partly explain a more posterior distribution of VPC activations during *recollection* tasks.

The finding of graded connectivity patterns within VPC seems at odds with cyto- and receptor-architectonic evidence indicating that this region is anatomically heterogeneous (Brodmann, 1911; Caspers et al., 2012). However, there is abundant evidence that the distribution of different cell types within specialized regions of the brain, and their respective connections, are generally more graded than discrete. Graded patterns of cell behavior and intermixed cell populations have been found in the visual cortex (Barone et al., 2000), auditory cortex (Castro and Kandler, 2010), MTL (Xiang and Brown, 1998), and frontal lobes (Barbas, 1986). Regarding VPC, using structural connectivity measures, Caspers et al. (2011) found that the middle VPC (approximately corresponding to TPJ) shared connection patterns of both rostral (posterior supramarginal) and caudal (angular) VPC areas, again suggesting that the functional/anatomical boundaries are not that clear, and rather show a rostro-caudal gradient in terms of structural connectivity. Thus, despite abundant evidence for modularity in the brain, available evidence suggests that the cytoarchitectonic boundaries between anatomical subregions, at least, within modules are more graded than sharp. Nevertheless, despite the fact that we found clear breaks of the gradients outside of VPC, some caveat is required regarding the effect of spatial smoothing on the reported gradients. Even though we used a minimal smoothing kernel of 4 mm with a 6-mm gradient gap, there could be some residual blurring effects.

The finding that connectivity differences between anterior and posterior VPC regions are more graded rather than sharp is important because it supports the development of over-arching models of VPC function, such as output buffer and evidence accumulator models (Wagner et al., 2005). The AtoM model is another exemplar of an overarching model, because it assumes that the area of VPC that encompasses both AG and SMG plays a an overarching role in bottom-up attention and that both anterior (SMG) and posterior (AG) VPC regions contribute to it. This model can parsimoniously account for VPC activations during both *attention-reorienting* and *recollection* tasks because both tasks depend on bottom-up attention. Importantly, this model is not incompatible with evidence that the spatial distribution of VPC activations is not identical for *attention-reorienting* and *recollection* tasks, because the specific locations of activations depend on several factors, including input/output differences. The current results fit well with this input/out hypothesis.

In addition to addressing a specific issue regarding the function of the VPC, this study also speaks to a general problem in interpreting functional neuroimaging data. Differences in regions of peak activation, and in functional connectivity with those regions, typically are interpreted as indicative of differences in function mediated by those regions (fractionation hypothesis). As we have argued, this need not be the case. These differences could just as easily reflect differences in applying a common (overarching) function to different domains, in our case perception and memory, about which information is conveyed via different pathways (Cabeza et al., 2012). Although we acknowledge that finding a graded response is not solid evidence for the “overarching function hypothesis” against the “fractionation hypothesis,” we hope that it is sufficiently compelling to encourage others to entertain both types of interpretations of functional neuroimaging data in other fields.

### Conflict of interest statement

The authors declare that the research was conducted in the absence of any commercial or financial relationships that could be construed as a potential conflict of interest.
